# Increasing socioeconomic gap between the young and old: temporal trends in health and overall deprivation in England by age, sex, urbanity and ethnicity, 2004–2015

**DOI:** 10.1136/jech-2017-209895

**Published:** 2018-03-19

**Authors:** Evangelos Kontopantelis, Mamas A Mamas, Harm van Marwijk, Iain Buchan, Andrew M Ryan, Tim Doran

**Affiliations:** 1 Faculty of Biology Medicine and Health, University of Manchester, Greater Manchester, UK; 2 NIHR School for Primary Care Research, University of Manchester, Greater Manchester, UK; 3 Centre for Prognosis Research, Institute for Primary Care and Health Sciences, Keele University, Stoke-on-Trent, UK; 4 Healthcare Research, Microsoft Research Cambridge, Cambridge, UK; 5 School of Public Health, University of Michigan, Ann Arbor, Michigan, USA; 6 Department of Health Sciences, University of York, York, UK

**Keywords:** deprivation, Index of Multiple Deprivation, IMD, health, age, sex, ethnicity, rurality, England

## Abstract

**Background:**

At a low geographical level, little is known about the associations between population characteristics and deprivation, and their trends, which would be directly affected by the house market, labour pressures and government policies. We describe temporal trends in health and overall deprivation in England by age, sex, urbanity and ethnicity.

**Methods:**

Repeated cross-sectional whole population study for England, 2004–2015, at a low geographical level (average 1500 residents). We calculated weighted medians of the Index of Multiple Deprivation (IMD) for each subgroup of interest.

**Results:**

Over time, we observed increases in relative deprivation for people aged under 30, and aged 30–59, while median deprivation decreased for those aged 60 or over. Subgroup analyses indicated that relative overall deprivation was consistently higher for young adults (aged 20–29) and infants (aged 0–4), with increases in deprivation for the latter. Levels of overall deprivation in 2004 greatly varied by ethnicity, with the lowest levels observed for White British and the highest for Blacks. Over time, small reductions were observed in the deprivation gap between White British and all other ethnic groups. Findings were consistent across overall IMD and its health and disability subdomain, but large regional variability was also observed.

**Conclusions:**

Government policies, the financial crisis of 2008, education funding and the increasing cost of houses relative to real wages are important parameters in interpreting our findings. Socioeconomic deprivation is an important determinant of health and the inequalities this work highlights may have significant implications for future fiscal and healthcare policy.

## Introduction

Numerous definitions exist for deprivation, while there has been a long and continuing debate about the domains and indices a complete measure of deprivation should encompass.^1 2^ There is almost universal consensus, however, that deprivation should be expressed in relative terms and hence a popular definition is that of a standard of living or quality of life that is below that enjoyed by the majority in the respective society, to a high enough extend to introduce hardship, little or no access to resources and underprivilege.^3^ Although health-related deprivation (eg, higher levels of morbidity and mortality) is strongly correlated with general deprivation, it is not fully explained by it, with well-known UK examples of this disparity being the ‘North-South divide’,^4 5^ and the ‘Glasgow effect’.^6^ Health-related deprivation can directly inform on the distribution of resources, as it does in the UK primary care through the global sum allocation formula.^7^ Currently, the formula only adjusts for standardised limited long-standing illness and the standardised mortality ratio for those under 65, however, and there have been formal calls for the use of a more complete and accurate measure of health-related deprivation.^8^ For universal care health systems, like the UK National Health Service, health inequalities are of paramount importance and need to inform policy and resource distribution, if they are to successfully act as a counterforce and facilitate social mobility.

Socioeconomic deprivation, primarily encompassing low income and little or no wealth or education, is also an important public health consideration which should influence the distribution of resources in universal healthcare systems.^9^ Socioeconomic deprivation is the most important of the social determinants of health, factors apart from medical care that can explain clinical outcomes.^10^ From a health policy point of view, taking steps to address socioeconomic inequalities may be as important as healthcare spending (or even more important), for improving population health. For example, only 10%–15% of preventable mortality in the USA was explained by medical care,^11^ while in the UK a large and expensive primary care pay-for-performance scheme with numerous quality indicators across a large number of chronic conditions was not associated with premature mortality,^12^ while socioeconomic deprivation was.^13^

A comprehensive 2010 government-led report on equality that investigated various aspects of deprivation across numerous demographic strata^14^ showed that household wealth varied by age (linearly increasing until the 55–64 age group, then declining) and ethnicity (highest for White British households), but gender inequalities were largely masked, although still present, at the household level. Income inequality was found to have reduced over time in households with at least one person aged 65, and, to a small extent, for women. The longitudinal comparison of income for ethnicity showed increases in earnings which moved or maintained some ethnic minorities to levels above White Britons. Despite the great value of this report, the data used are now almost 10 years old and predate the 2008 financial crisis. A more recent report highlighted that absolute rates of poverty have been decreasing for all age groups, but the largest decreases were observed for pensioners, who overtook working age non-parents after the 2008 financial crisis and by 2014–2015 had the lowest rates among all groups.^15^ Within this report, information on ethnicity was limited, but nevertheless it was shown that households with at least one non-white member reported higher rates of material deprivation.

The relevance of deprivation, both socioeconomic and health related, to healthcare policy and public health highlights the importance of investigating shifting time-trends in relative deprivation across population strata and over space, allowing for the evaluation of existing policies or identifying the need for new interventions to address inequalities. Although the Index of Multiple Deprivation (IMD) is standardised in each time point and does not allow for the investigation of absolute changes,^16–18^ it is possible to assess relative changes over time. At a low geographical level, little is known about the associations between population characteristics and relative deprivation, and their trends, which would be directly affected by the house market, labour pressures and government policies. The aim of this paper was to quantify the temporal trends in overall and health-related deprivation from 2004 to 2015, by location urbanity and population age, sex and ethnicity. Primarily we were interested to assess whether there was a deprivation location gap between the young and old, and how it changed over time, and similarly for various ethnic groups compared with White Britons.

## Methods

Details about the methods and the data sources, in relation to the English IMD and the 2011 census data, which were collected and analysed at the lower super output area (LSOA) level, are presented in online supplementary appendix 1 and elsewhere.^19^ The IMD quantifies relative deprivation across seven domains: income, employment, education and skills, health and disability, crime, barriers to housing and services, and living environment.^20^ Details about each underlying indicator included in the domains are provided in online supplementary appendix 2, table B1. In the health deprivation domain, information is aggregated on years of potential life lost, illness and disability, acute morbidity, and mood and anxiety disorders. The census and deprivation information was available at the LSOA level, a low-level geography designed to contain 1500 inhabitants on average (not available at the person level, only as regional aggregates). LSOAs were organised into 10 regions to allow for comparisons within England, based on the 2006 restructuring of Strategic Health Authorities: North East, North West, Yorkshire & the Humber, East Midlands, West Midlands, East of England, London, South East Coast, South Central and South West.^21^

10.1136/jech-2017-209895.supp1 Supplementary file 1

10.1136/jech-2017-209895.supp2 Supplementary file 2

### Analyses

The outcomes of interest were overall deprivation as measured by the English IMD and the health domain of the English IMD, for 2004, 2007, 2010 and 2015.

To assess temporal trends in the deprivation outcomes of interest by age, sex and ethnicity, between 2004 and 2015, we calculated population weighted deprivation medians over time for each population group of interest using the *epctile* command in Stata.^22^ Assuming a categorical variable of interest X with n categories (eg, age group), and a continuous outcome Y (eg, IMD), with both variables measurable across N units (eg, LSOAs), we can calculate a median for Y weighted on $X_{j}$ (eg, where j is the population aged 0–29). More specifically, for N ordered elements $Y_{1},Y_{2}\ldots Y_{N}$ with weights $X_{j1},X_{j2}\ldots X_{jN}$ the weighted median is the element $Y_{k}$ satisfying $\sum_{i=1}^{k-1}X_{ji}\leq \frac{1}{2}\sum_{i=1}^{N}X_{ji}$ and $\sum_{i=k+1}^{N}X_{ji}\leq \frac{1}{2}\sum_{i=1}^{N}X_{ji}$.

Age was categorised into three groups: aged 0–29, 30–59 and 60 or over. Additional analyses were conducted on age subgroups for the 0–29 group: 0–4, 5–14, 15–19 and 20–29. Although numerous categories exist for ethnicity, we aggregated smaller ethnic groups for easier reporting, and the final categorisation was: White British, White Other, Asian, Black, and Mixed race and Other. For urbanity, each LSOA is classified as rural or urban. For age and sex subgroups, we also considered decomposing the change in the subgroup IMD from 2004 to 2015, into changes in the distribution of the population and distribution of the IMD at the LSOA level (online supplementary appendix 1).

Results are reported for the whole of England and each of the 10 regions. All analyses were executed with Stata V.14.1. Because of the size of the data set, effectively the whole of England, statistical significance is largely irrelevant; all comparisons would be statistically significant and thus we try to focus on effect sizes where possible.

## Results

**Figure 1:**
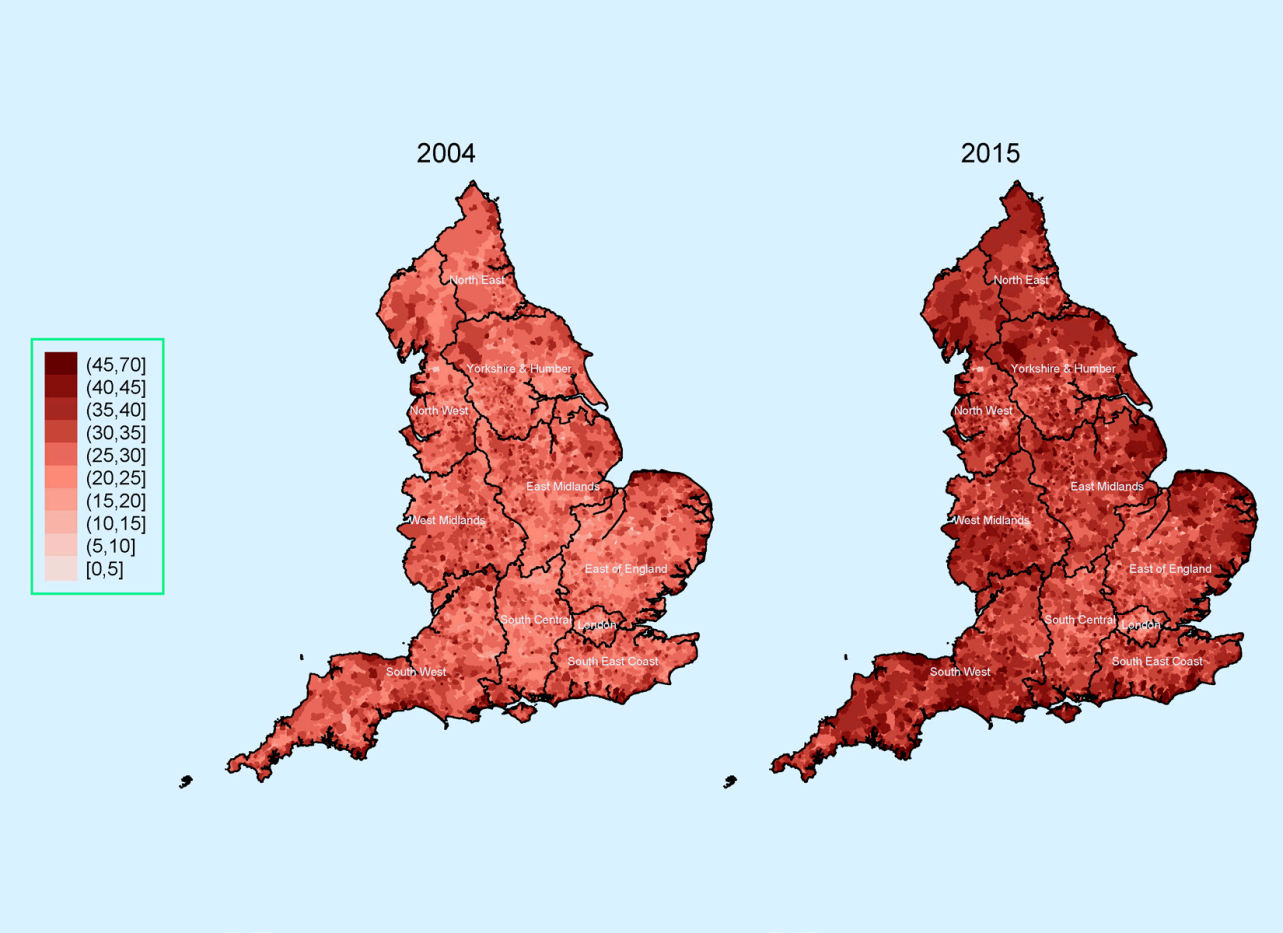
Percentage of people aged 60 or over in England as the lower super output area (LSOA) level, 2004 (left) and 2015 (right)*†. *The mean percentage of people aged 60 or over (across all LSOAs) rose from 21.1% in 2004 to 23.9% in 2015. †The dark lines indicate county boundaries within each region. The colour version of this figure is available online.

**Figure 2:**
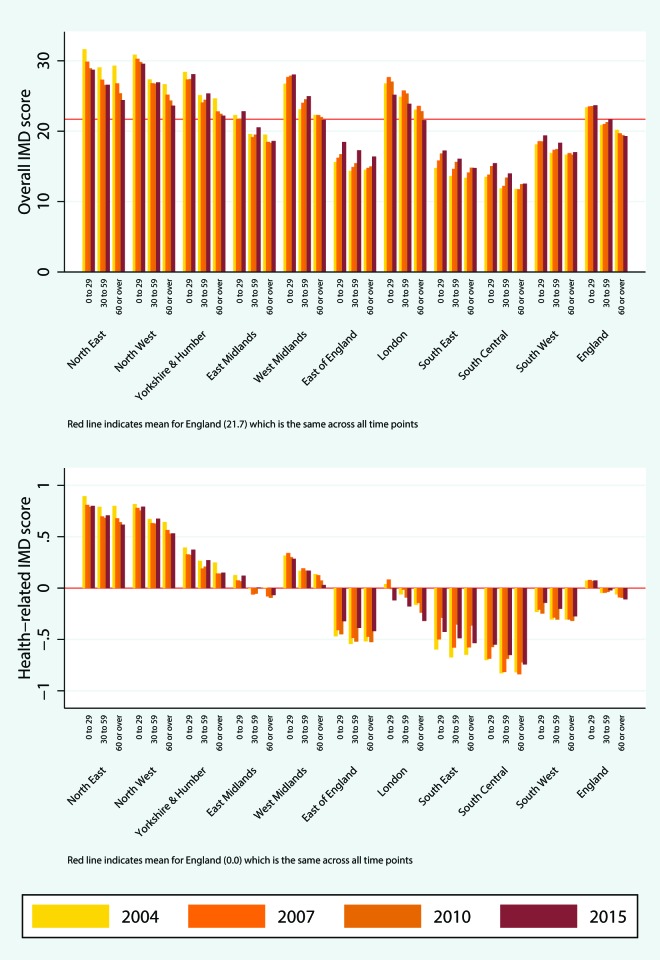
Median overall deprivation (top) and health domain deprivation (bottom) by age group and region, 2004–2015. IMD, Index of Multiple Deprivation. The colour version of this figure is available online.

**Figure 3:**
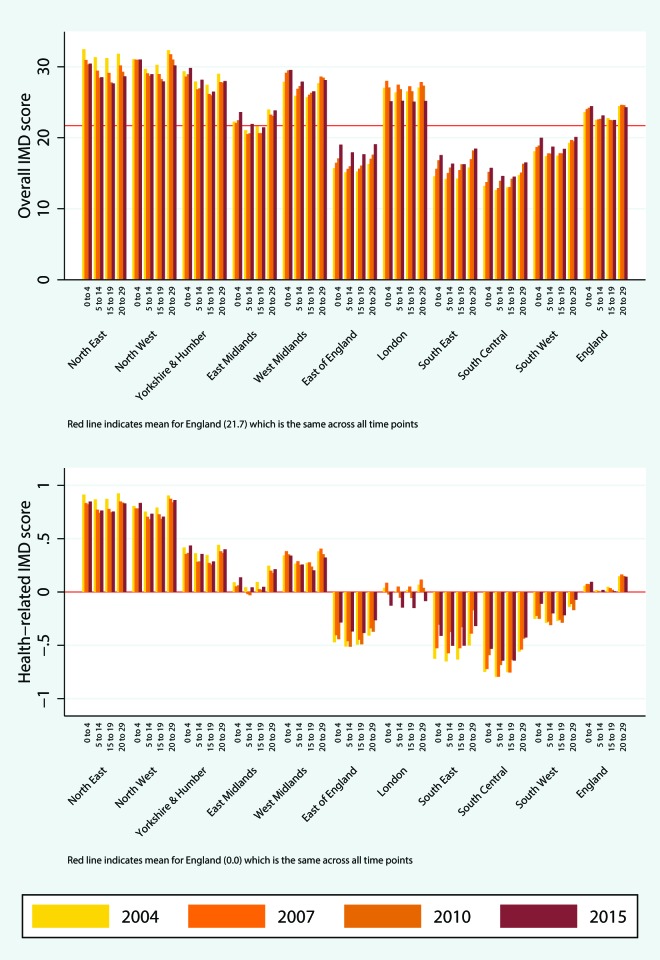
Median overall deprivation (top) and health domain deprivation (bottom) by under 30 age group and region, 2004–2015. IMD, Index of Multiple Deprivation. The colour version of this figure is available online.

**Figure 4:**
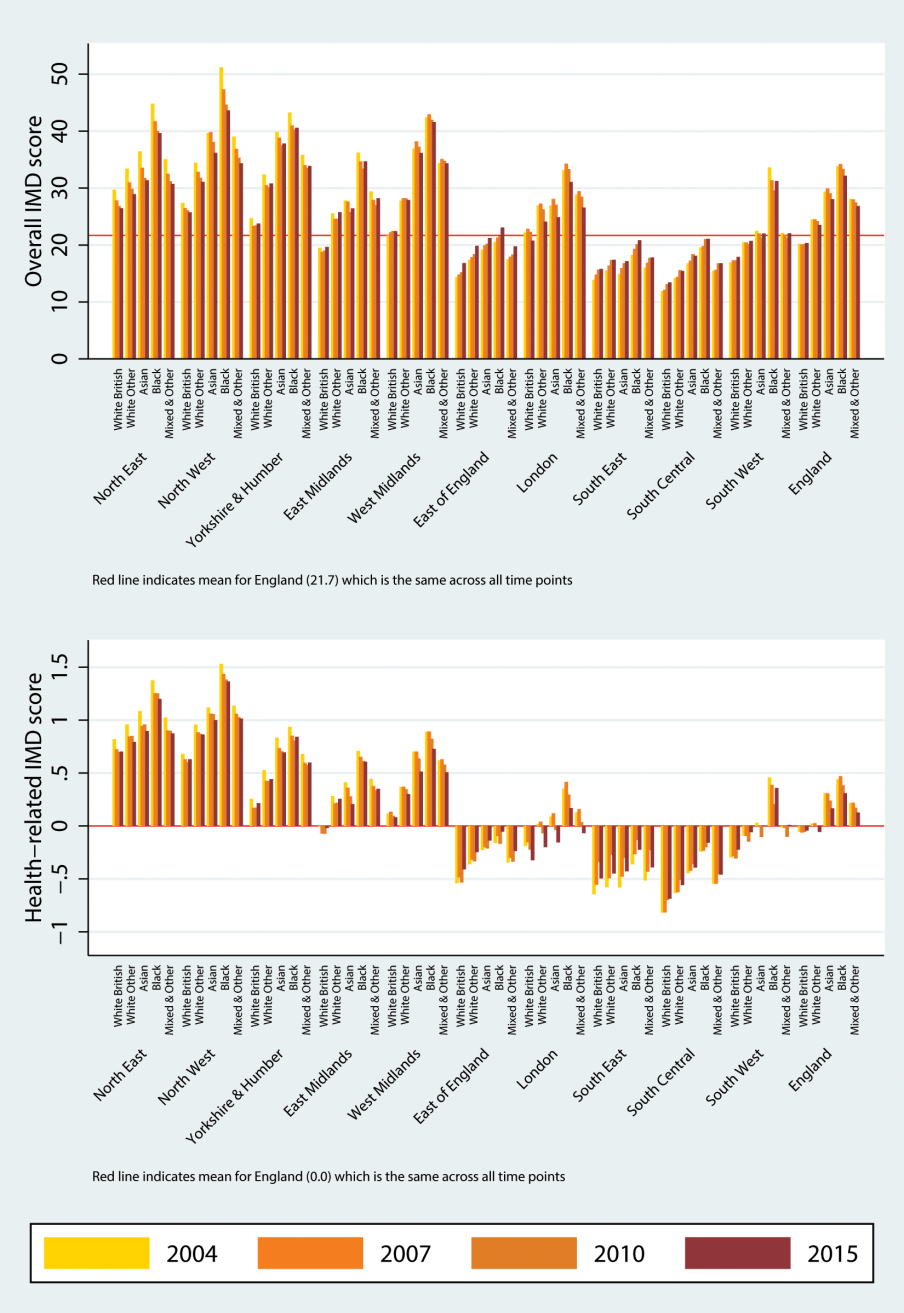
Median overall deprivation (top) and health domain deprivation (bottom) by ethnicity and region, 2004–2015. IMD, Index of Multiple Deprivation. The colour version of this figure is available online.

The characteristics of England and its 10 regions in terms of age, sex, ethnicity and rurality are presented in table 1. The different characteristics of London, compared with the rest of the country, are well known, with London being by far the most multiethnic region (online supplementary appendix 2, figure B1). A spatial representation of the percentage of people aged 60 or over at the LSOA level is presented in figure 1, where high within-region and between-region variability can be observed. For the whole of England, deprivation levels appear relatively stable over time, across all covariates of interest (figures 2–4; online supplementary appendix 1, figures A1 and A2). The distributions, summary statistics and centiles for both outcomes are provided in online supplementary appendix 2 (figures B2 and B3, tables B2 and B3) to aid the interpretation of the findings. Gender and rurality deprivation is presented and discussed in online supplementary appendix 1. We observed no differences across gender for overall or health-related deprivation in location deprivation (online supplementary figure A1). Overall and health-related deprivation in rural areas remained much lower than in urban areas, but post-2010 we saw modest increases in their overall deprivation, primarily driven by increases in West Midlands, East England and the South West (online supplementary figure A2).

**Table 1:** 2011 census ethnicity and rurality and 2015 predictions for age and sex, for England and by region

	England	North East	North West	Yorkshire & the Humber	East Midlands	West Midlands	East of England	London	South East	South Central	South West
LSOAs	32 844	1657	4497	3317	2774	3487	3614	4835	2773	2609	3281
People	54 786 327	2 624 621	7 173 835	5 390 576	4 677 038	5 751 000	6 076 451	8 673 713	4 635 616	4 312 297	5 471 180
Age (%)											
0–29	37.2	36.2	37.1	37.7	36.5	38.0	35.7	40.9	35.1	36.8	34.5
30–59	39.8	38.9	39.2	38.8	39.1	38.5	39.7	43.6	39.5	40.3	38.2
60 or over	23.0	24.9	23.6	23.5	24.4	23.5	24.6	15.5	25.4	22.9	27.3
Sex (%)											
Female	50.7	51.0	50.7	50.7	50.6	50.5	50.7	50.3	51.0	50.5	50.9
Ethnicity (%)											
White British	77.2	92.6	85.6	84.1	82.8	77.1	82.1	42.3	84.0	80.3	88.8
White Other	5.5	1.7	3.1	3.0	3.7	3.5	5.3	14.0	5.2	5.3	3.5
Asian	7.6	2.8	6.1	7.2	6.3	10.5	4.6	17.4	3.7	6.5	1.9
Black	3.4	0.5	1.4	1.5	1.7	3.2	1.9	12.6	1.1	2	0.9
Mixed or Other	3.2	1.3	2.2	2.3	2.4	3.2	2.3	7.9	2.3	2.6	1.6
Urbanity (%)											
Rural	17.0	17.6	9.8	16.5	25.4	14.8	28.3	0.2	20.1	19.8	30.2

LSOA, lower super output area.

### Deprivation across age groups

We observed diverging trends for the three age trends for both outcomes, and we now focus on overall deprivation only. Against a national median of overall deprivation close to 17, in 2004 the weighted median for people aged under 30 was 18.9 (54th centile) and increased little over time to 19.9 (57th centile) in 2015. A larger increase was observed for those aged 30–59, from 16.2 (48th centile) in 2004 to 17.6 (51st centile) in 2015. For those aged 60 or over the trend was reversed with a median deprivation of 15.6 (46th centile) in 2004 gradually declining to 15.1 (44th centile) in 2015. The cumulative difference in difference of 1.8 (95% CI 1.6 to 2.0) between the two older age groups is small but not negligible and roughly corresponds to a difference of just over five percentile points in overall deprivation, using the 2015 distribution and the 50th centile as a starting point. Changes between 2004 and 2015 for each age group and by region are presented in figure 2. There we observe that within each region the temporal changes for those aged 60 or over are more beneficial compared with the other two age groups. In other words, when deprivation for a region increased between 2004 and 2015, the eldest group was the one least affected and when it decreased it was the age group that benefited the most, on average.

Decomposing the change in IMD for age subgroups into changes in the distributions of the population and IMD, from 2004 to 2015, showed that, overall, IMD distributional changes account for a small percentage of changes in deprivation for age subgroups, with the exception of those aged 0–29, the least mobile subgroup (online supplementary appendix 1). The analyses for the under 30 subgroups (aged 0–4, 5–14, 15–19 and 20–29) indicated that overall deprivation is consistently higher for infants (0–4) and young adults (20–29). For infants, there was also a small increase over time, with the median overall deprivation of 18.9 (54th centile) in 2004 increasing to 20.5 (58th centile) in 2015 (figure 3).

### Deprivation across ethnic groups

Levels of overall deprivation in 2004 greatly varied by ethnicity, with the lowest levels observed for White British and the highest for Blacks (figure 4). In 2004, for example, median overall deprivation for Blacks in the North West was more than double of what was observed for White British, with the gap narrowing by 2015. Over time, overall and health domain deprivation very slightly increased for White Britons, while small reductions were observed for all other ethnic groups. The largest reductions in both outcomes were observed for Blacks and Asians. For example, for Blacks, median overall deprivation dropped from 32.9 (79th centile) in 2004 to 31.5 (78th centile) in 2015. For the other ethnic groups, median overall deprivation levels in 2015 were 15.9 (47th centile) for White Britons, 20.7 (59th centile) for White Other and 26.0 (69th centile) for Asians. We also observed great regional variability in ethnic differences. For example, the largest differences between the least affluent (Blacks) and most affluent groups (White British) were seen in the North West and West Midlands. By contrast, the smallest variability in ethnic deprivation was observed in the East of England, the South East and South Central.

## Discussion

Our work for the first time describes important temporal changes in deprivation over a decade in England, with important differences described in different geographical locations and among different groups within society stratified by gender, age and ethnicity. The inequalities that our work highlights among different groups within society may have significant implications for future healthcare policy.

England has an increasingly ageing population: the percentage of people aged 60 or over rose from 21.1% in 2004 to 23.9% in 2015. Over the same time period, we observed diverging trends of overall deprivation for different age groups. On average, people aged 60 or over not only live in less deprived areas, but the inequality gaps between those aged 60 or over and the other two age groups (0–29 and 30–59 age groups) have increased over time. Within the 0–29 age group, we observed increases in overall deprivation for all subgroups, the highest for infants (aged 0–4) and children (aged 5–14), especially after 2010 for the latter.

All ethnic groups generally live in more deprived locations compared with White Britons. On average, Blacks lived in the most deprived locations, followed by Asians, Mixed race and Other, and White Other. However, deprivation for ethnic groups has improved gradually over time for overall and health-related deprivation. Large regional variations were observed in the inequality gaps across ethnic groups, with the largest gaps in North West (health-related deprivation) and West Midlands (overall deprivation).

### Strengths and limitations of the study

The major strength of this study is its use of national data, covering the whole of England from 2004 to 2015, allowing for numerous comparisons across various subpopulations and regions.

Some limitations exist. First, there were some minor changes in the underlying deprivation indicators over time, which might have influenced our change estimates. However, the trends we observe persist between 2007 and 2010 where there were no changes at all to the measures, while the health deprivation domain has remained unchanged across the whole time period. Second, the IMD and each of each domains are normalised and standardised at each time point, hence the measure cannot account for absolute longitudinal change for each group of interest, only relative improvement or deterioration.^23^ Third, the population data we used are based on decennial census information. For population levels within each LSOA, and their age and sex structures, we used the Office for National Statistics annual predictions for the years of interest (2004, 2007, 2010 and 2015) based on 2001 and 2011 census data. For rurality, we used the classification based on the 2011 census, and we assumed it was static over the 11-year study period, which may be valid considering the very strong correlation (≈1) between the 2001 and 2011 classifications. However, that assumption does not necessarily stand for ethnicity, for which information was also based on the 2011 census, despite the large correlations between 2001 and 2011 data. Therefore, some of the trends regarding ethnicity may be explained by internal or external migration over time and resulting changes in ethnic distributions within LSOAs, which we cannot quantify. Fourth, some of the underlying indicators in the IMD are not necessarily independent of the variables that defined the subgroups we investigated. For example, social security benefits are not relevant for all ages and will not be equally distributed across age groups, so by definition the IMD will be higher for people of working age, on average, ‘all else being equal’. Nevertheless, we would argue that even when ‘all else is equal’, a higher proportion of people on benefits should indicate higher deprivation, while we are primarily focused on changes between 2004 and 2015 for each subgroup, rather than between-group comparisons, where this is less of a concern. Fifth, the subgroup deprivation scores we calculated are estimates under certain distributional assumptions, but there is no alternative to this since IMD scores at the LSOA level are not reported for population subgroups or at the individual level.

### Findings

Social inequality is known not to be consistent across the life course,^24^ with greater mortality risks for most ages in more deprived areas, except during late adolescence.^25^ This work has identified diverging trends of overall deprivation for the younger and older age groups, with the over 60s living in less deprived areas and improving their relative position over time. Our findings are in agreement with previous work which indicated that poverty rates have reduced the most for pensioners and are now the lowest among all age groups.^15^ These trends are very likely driven by government policies and changes in the housing and labour market. The housing market is arguably the most important parameter, with the average house in England and Wales costing 7.6 times the average annual salary in 2016, up from 3.6 times in 1997.^26^ Considering the housing market is a self-reinforcing driver of wealth inequality,^27^ the large increases in house prices over a relatively short period of time have provided a large advantage to the older generation (for whom it was much cheaper to get on the property ladder, earlier). In addition, real pay fell sharply after the 2008 crisis, and although it somewhat recovered between 2014 and 2016, the recovery has been negated post-Brexit.^28^ This imbalance between house prices, driven by supply and demand (with recent policy aiming to drive down demand by targeting the buy-to-let market),^29^ and real pay, driven by global pressures and pension deficits,^30^ has put considerable pressure on the younger generation. This pressure does not seem to be limited to those of working age, but applies to infants and children, possibly indicating rising costs for parents (£231 843 on average to raise a child, up 65% from 2003).^31^

In terms of decomposing the changes in deprivations over time for these age subgroups, this was primarily attributed to changes in the population distribution (ie, more deprived LSOAs becoming more populous over time, and the age distribution across LSOAs changing either through mobility or ageing) and only to a very small extent due to changes in the IMD distribution, with the exception of those aged 0–29 (online supplementary appendix 1). This is in agreement with previous work, where very strong correlations were identified over time for IMD at the LSOA level,^19^ while the influence of population migration on inequalities is known to vary by age.^32^ Deprivation immobility is a major concern and has been linked to very high levels of premature mortality.^33^

The patterns of overall and health-related deprivation by ethnic group indicate that large gaps exist compared with White Britons, in agreement with other work,^34^ and these gaps are much wider in the North of England than in the South. These regional differences are not explained by the higher levels of deprivation in the North (which allow for more variability), with striking overall deprivation gaps for postindustrial regions like the North West and West Midlands. The contrast between North and South in terms of premature mortality is well known,^4^ and we observed similar large regional differences for health-related deprivation (which includes premature mortality, illness and disability, acute morbidity, and mood and anxiety disorders) across all ethnic groups. Regarding the improving standing of all ethnic groups over time, both in overall and health-related deprivation and in agreement with previous work,^35^ it can be at least partly explained by an expectation of increased earnings for migrants (relative to the natives) over time. The longer migrants have spent in the UK, the more likely it is they will have reached or overcome the average salary for natives, with the average time to achieve salary parity estimated to be 20 years for men but only 5 for women.^36^ In addition, relative pay of migrants to natives has been consistently higher for women since 1987 and increased to parity for men by 2003.^36^ This is in agreement with observed desegregation of ethnic minorities from 1991 to 2011, with increased residential mixing between each ethnic group,^37^ and a spreading out of ethnic diversity from urban centres towards areas traditionally less diverse and historically more affluent.^38^ Transitions across deprivation quantiles for ethnic minorities can contribute to changing health gradients, and movement within the middle deprivation quintiles may be particularly important in terms of the contribution to changing health gradients.^39^

For sex, there were no differences in overall or health deprivation at any point in time, which is not surprising. It is known that socioeconomic disparities at the individual level between sexes are masked at the household or geographical area level.^14^ Finally, we observed a gap in health outcomes between urban and rural areas, which have closed little over time. In this context, the higher pay per patient in general practices serving rural areas may be relevant.^40^

## Conclusions

Relative overall deprivation trends at a low geographical area are diverging for different age groups, with those aged 60 or over living in less deprived areas and improving their standing over time, compared with other age groups. Government policies, the financial crisis of 2008, how education is being financed and the increasing cost of houses relative to real wages are important parameters in interpreting this effect. Average overall deprivation levels for infants and children increased, which may partly reflect the increasing costs of raising children. Overall and health-related deprivation was consistently higher for all ethnic groups than for White Britons, with small reductions in the differences over time.

Socioeconomic deprivation is an important determinant of health and healthcare need. The important inequalities that our work highlights among different groups within English society may have significant implications for future fiscal and healthcare policy. Healthcare policy should aim to prioritise deprived areas, perhaps by more successfully distributing funds according to local healthcare need. Fiscal policy should take into account the increasing resource gap between the young and the old and aim to deliver a fairer society.

**What is already known on this subject**

- Socioeconomic deprivation is an important public health consideration which should influence the distribution of resources in universal healthcare systems.
- Absolute poverty rates have consistently decreased over time for all population groups in England, and by 2014–2015 pensioners had the lowest rates.
- Levels of deprivation are higher for ethnic minorities.
- However, at a low geographical level, little is known about the associations between population characteristics and deprivation, and their trends over time.

**What this study adds**

- Between 2004 and 2015, relative overall deprivation for people aged under 30 and for those aged 30–59 increased, while it decreased for those aged 60 or over.
- Relative deprivation was consistently higher for young adults (aged 20–29) and infants (aged 0–4), with increases in deprivation for the latter.
- Over time, small reductions were observed in the relative deprivation gap between White British and all other ethnic groups.
- Fiscal policy should take into account the increasing resource gap between the young and the old and aim to deliver a fairer society.
