# Supplementary material for: Increasing socioeconomic gap between the young and old: temporal trends in health and overall deprivation in England by age, sex, urbanity and ethnicity, 2004–2015
Source: J Epidemiol Community Health. 2018 Mar 19;72(7):636–44. doi: 10.1136/jech-2017-209895 (PMC6031281; doi:10.1136/jech-2017-209895)

Online appendix 1 for: "The increasing socio-economic gap between the young and old: temporal trends in health and overall deprivation in England by age, sex, urbanity and ethnicity, 2004 to 2015"

Expanded methods

English Indices of Deprivation

In the UK, the most complete and widely used approach to quantify deprivation and affluence is through the Indices of Deprivation, and their aggregate score. Separate deprivation measures exist for UK countries, with varying degrees of similarity. The English Index of Multiple Deprivation (IMD) quantifies relative deprivation across seven domains: income, employment, education and skills, health and disability, crime, barriers to housing and services, and living environment.[1] The overall IMD is calculated as a weighted mean across the seven domains, with income and employment deprivation given the largest weight (22.5% each), followed by health and education deprivation (13.5% each), and with the other three domains given equal weights (9.3%). Details about each underlying indicator included in the domains are provided in online appendix 2 Table B1 and elsewhere.[2] In the health deprivation domain, information is aggregated on years of potential life lost, illness and disability, acute morbidity and mood and anxiety disorders. Although the underlying indicators can inform on longitudinal trends, the domain scores and the overall IMD score are transformed and centred, and therefore cannot account for longitudinal improvement or deterioration at the country level. However, the aggregate scores can be used to evaluate longitudinal regional changes and country-level distributional changes. The English IMD was first calculated and reported for 2004, under an area-based model, and since then updates have followed for 2007, 2010 and 2015. Although small changes to the indices have been implemented over time, comparability has been maintained since the methods used are the same and no major changes have been considered necessary.[1]

2011 Census data

In the 2011 decennial UK census, information was collected on various population characteristics, including age, sex and ethnicity.[3] We focused on these three variables for a number of reasons: they are relevant to policy; they are independent to deprivation unlike many socio-economic variables in the census; there is reasonable variability in them at a low geographical (and most granular) level; age and sex predictions exist for all years between 2001 and 2015 (based on 2001 and 2011 census data),[4] while we expect ethnicity to not fluctuate much over the time period of interest. A fourth variable of interest, not directly extracted from the 2011 census but computed using 2011 census data, was urban and rural classification of low UK geographies.[5] We used a rural vs urban dichotomy for simplicity, with settlements with 10,000 people or more defined as urban, although the measure is much more refined and includes six rural categories (town & fringe; villages; hamlets & isolated dwellings – all three by sparse and not sparse classification) and four urban (major conurbation, not sparse; minor conurbation, not sparse; city & town, not sparse, and city & town in a sparse setting).[6]

Lower Super Output Areas

The census and deprivation information was available at the lower super output area (LSOA) level, a low-level geography designed to contain 1500 inhabitants on average. Following the 2011 census, English LSOAs were re-organised into 32,844 units (from 32,482 after the 2001 census) to better reflect population changes, mainly increases.[7] Only 2.5% of English LSOAs merged, split or underwent a more complicated change.[8] To assign pre-2011 data (generally reported using 2001 LSOAs) to the 2011 LSOA structure, we created a weighted means algorithm. Spatial coordinates for the 2011 LSOAs were obtained from the ONS open geography portal.[9] We used digital vector boundaries generalised to 20 meters and clipped to the coastline to reduce size and improve visualisation. Finally, LSOAs were organised into 10 regions to allow for comparisons within England, based on the 2006 restructuring of Strategic Health Authorities: North East, North West, Yorkshire & the Humber, East Midlands, West Midlands, East of England, London, South East Coast, South Central and South West.[10]

Analyses

Since data for ethnicity and urbanity were only available for 2011, over the study period, all analyses necessarily assumed there were no changes in these two variables over time. Since this is a potential limitation of the analyses and changes in deprivation could be driven by changes in these variables, we evaluated the changes in ethnicity and urbanity since the 2001 census. Very high correlations would allow us to argue that there is very little change over time in these two characteristics.

In out analyses we used weighted medians to better account for the skewed distribution of the IMD. However, if we had used the weighted median, the change in deprivation could have been decomposed and attributed to changes in the population distribution or changes in the IMD, over time. Assume $Y_{x\,t}$ the deprivation for population subgroup of interest x for time t, then the weighted mean of deprivation would be $Y_{x\,t} = \sum_{i=1}^{N} Y_{t\,i} \cdot S_{x\,t\,i}$, where Y_{ti} the IMD in LSOA i for time t and S_{xti} the proportion of group x in LSOA i for time t. The change in deprivation between time points $t-1$ and t can be decomposed as $Y_{x\,t} - Y_{x\,t-1} = \sum_{i=1}^{N} Y_{t\,i}\,(S_{x\,t\,i} - S_{x\,t-1\,i}) + \sum_{i=1}^{N} S_{x\,t-1\,i}(Y_{t\,i} - Y_{t-1\,i})$. Therefore, the change in mean deprivation for a particular age group is decomposed into population changes (including, changes in the population distribution within the LSOA due to moving and/or ageing, and changes in the total population within the LSOA) and changes in the IMD distribution. We used this approach to decompose the change in IMD for the three age groups of interest only (0-29, 30-59 and 60 or over). For sex, there was no overall change to decompose, and for ethnicity and urbanity we necessarily assumed no changes in their distributions over time.

Additional results

The median for overall deprivation slightly increased from 17.0 in 2004 to 17.4 in 2015, reflecting a small change in its distribution over time; a less elongated high deprivation tail, which moved the median closer to the constant mean of 21.7. The correlations between 2001 and 2011 for ethnicity and rurality, the two variables we necessarily assumed were static over the study period, were very

high at the national level. Tetrachoric rho for rurality was 0.997, while Pearson's rho for all ethnic groups were 0.9 or higher. When analysing by region, the picture was the same for rurality with all tetrachoric rhos very close to 1. For ethnicity, although most associations were close to 0.9 or above, there were a few that were in the 0.6 to 0.8 range (mainly for the Other White group, reflecting EU immigration between 2001 and 2011). The distributions, summary statistics and centiles for both outcomes are provided in this online appendix 1 (Figures A2 & A3, Tables A2 & A3) to aid the interpretation of the findings.

In terms of the decomposition of change in IMD between 2004 and 2015 for age subgroups, those aged 30-59 were the least affected by deprivation changes and were the most likely to be affected by population changes (people getting older, LSOA population sizes changing, people in this age group moving to other LSOAs). Unsurprisingly, the 0-29 group is least affected by population changes (since less likely to relocate). Overall, IMD changes account for a relatively modest amount of change in deprivation for age subgroups, from 2004 to 2015. This is in agreement with recent work, where we observed very strong correlation in the IMD between 2004 and 2015 (ρ=0.94 for overall IMD; ρ=0.92 for health-related IMD).[2]

**Age group**	**Population change***	**Deprivation change**
0-29	51.9%	48.1%
30-59	91.6%	8.4%
60 or over	81.6%	18.4%

* including changes in LSOA sizes and changes in age-group distributions within LSOAs due to ageing or relocating

Deprivation across gender

Over time, both overall and health-specific deprivation levels for men and women were almost identical, across all regions (Figure B1). As expected, due to the increase of the overall IMD median across LSOAs over time, we observed a small increase in the median across both sexes (17.0 in 2004 to 17.8 in 2015 for males; 17.0 in 2004 to 17.6 in 2015 for females). Changes in deprivation levels within a region were the same for both sexes.

These increases cannot be fully explained by the increases in the national median. A second reason behind the increase is that the most deprived areas have become more populous over time, compared to the more affluent ones. Focusing on the bottom 10% and top 10% if LSOAs in terms of overall deprivation we see increases in the populations of both groups, from 2004 to 2015, but the increase in the most deprived LSOAs was greater.

Deprivation across rurality

Rural areas had lower overall deprivation and health domain deprivation, for all time points and for all English regions (Figure B2). For the whole of England, overall and health domain deprivation in urban areas very slightly decreased over time. On the other hand, for rural areas, there was a noticeable but small increase in both outcomes, over time. As for ethnicity, the most striking

observation was the within-region variability and how different patterns were observed between the North and the South of the country. The largest within-regional differences between urban and rural areas were seen for the North West, Yorkshire & the Humber, and the West Midlands.

Finally, a large North-South variation also exists in differences between urban and rural areas, for both overall and health-related deprivation. There is a deprivation contrast between post-industrial urban centres of the North and their surrounding rural areas. The lower overall deprivation scores for rural areas were expected, mainly due to the Living Environment domain within the measure and its underlying indicators. However, we see even larger differences (in terms of corresponding centile on the distribution) for health-related deprivation, which indicates that there exists a real gap in health outcomes between urban and rural areas, which have closed little over time. In this context, the higher pay per patient in general practices serving rural areas may be relevant.[11]

References

1. Communities and Local Government. The English Indices of Deprivation 2015: Technical Report. Government DfCaL, 2015. https://www.gov.uk/government/publications/english-indices-of-deprivation-2015-technical-report
2. Kontopantelis E, Mamas M, van Marwijk H, Ryan AM, Buchan I, Ashcroft DM, et al. Geographical epidemiology of health and overall deprivation in England, its changes and persistence from 2004 to 2015. *J Epidemiol Community Health* 2017.
3. Office of National Statistics. Census 2011 Table Links. https://www.nomisweb.co.uk/census/2011/quick_statistics
4. Office for National Statistics. Super Output Area mid-year population estimates for England and Wales, Mid-2011 (Census Based). 2016. http://www.ons.gov.uk/ons/publications/re-reference-tables.html?edition=tcm%3A77-285629
5. Bibby P, Shepherd J. Developing a new classification of urban and rural areas for policy purposes–the methodology. *London: DEFRA* 2004.
6. Bibby P, Brindley P. Urban and rural area definitions for policy purposes in England and Wales: Methodology. *Government Statistical Service, London* 2013.
7. Office for National Statistics. Changes to Output Areas and Super Output Areas in England and Wales, 2001 to 2011. ONS, 2012:13. http://www.ons.gov.uk/ons/guide-method/geography/products/census/report--changes-to-output-areas-and-super-output-areas-in-england-and-wales--2001-to-2011.pdf
8. Office for National Statistics. Lower Layer Super Output Area (2001) to Lower Layer Super Output Area (2011) to Local Authority District (2011) Lookup in England and Wales. 2016. http://geoportal.statistics.gov.uk/datasets/6d3b1fc88b284a9bb9d4827530b16da4_0
9. Office for National Statistics. Open Geography Portal. 2016. http://geoportal.statistics.gov.uk/
10. Department of Health. Strategic Health Authority Configurations. https://web.archive.org/web/20070205232558/http://www.dh.gov.uk/assetRoot/04/13/37/60/04133760.pdf
11. Kontopantelis E, Mamas M, van Marwijk H, Ryan AM, Guthrie B, Doran T. Chronic morbidity, deprivation and primary medical care spending in England in 2015-16: a spatial analysis. *BMC Medicine* 2018.

Figure A1: Median overall deprivation (top) & health domain deprivation (bottom) by sex and region, 2004 to 2015

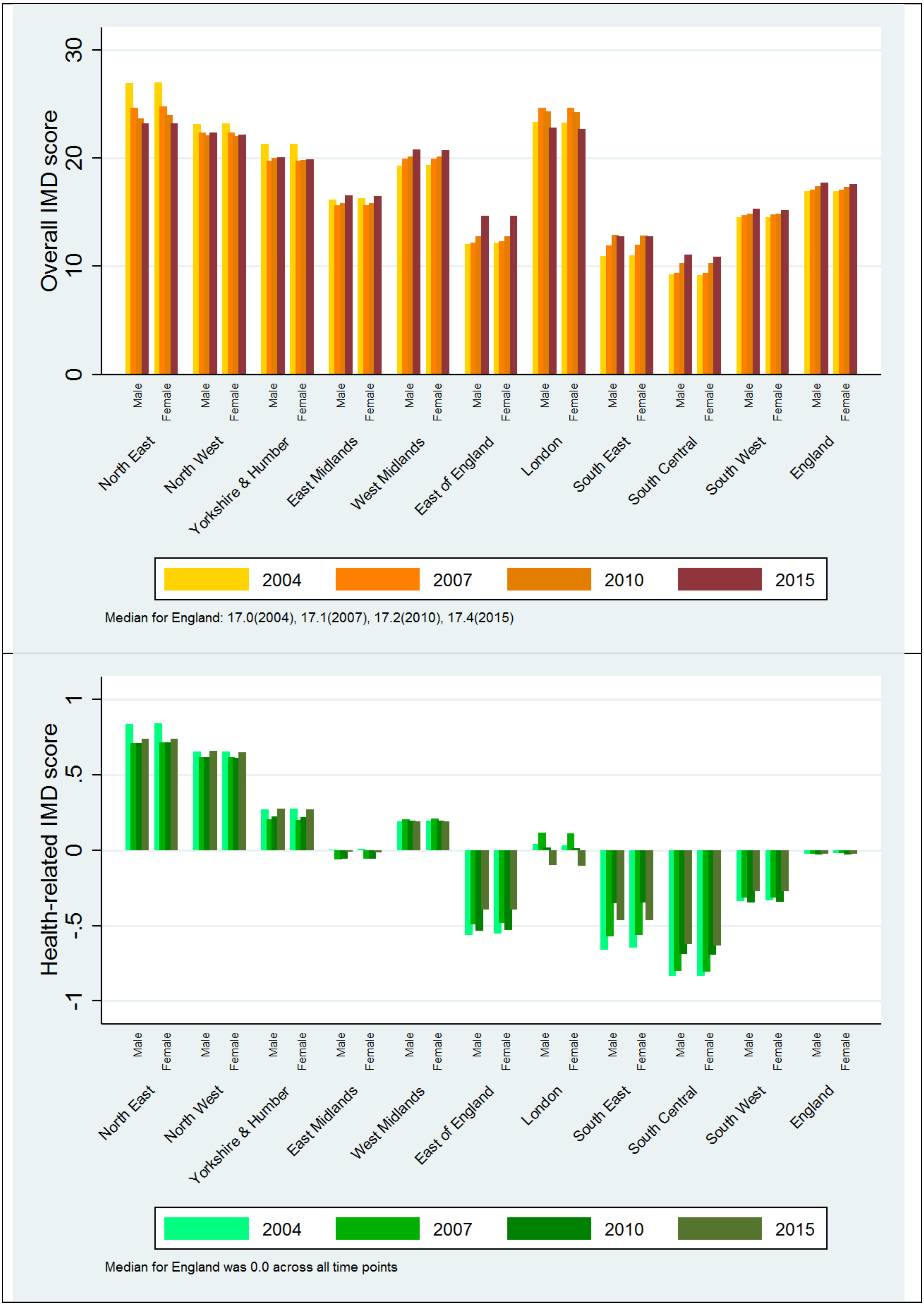

Figure A2: Median overall deprivation (top) & health domain deprivation (bottom) by urbanity and region, 2004 to 2015

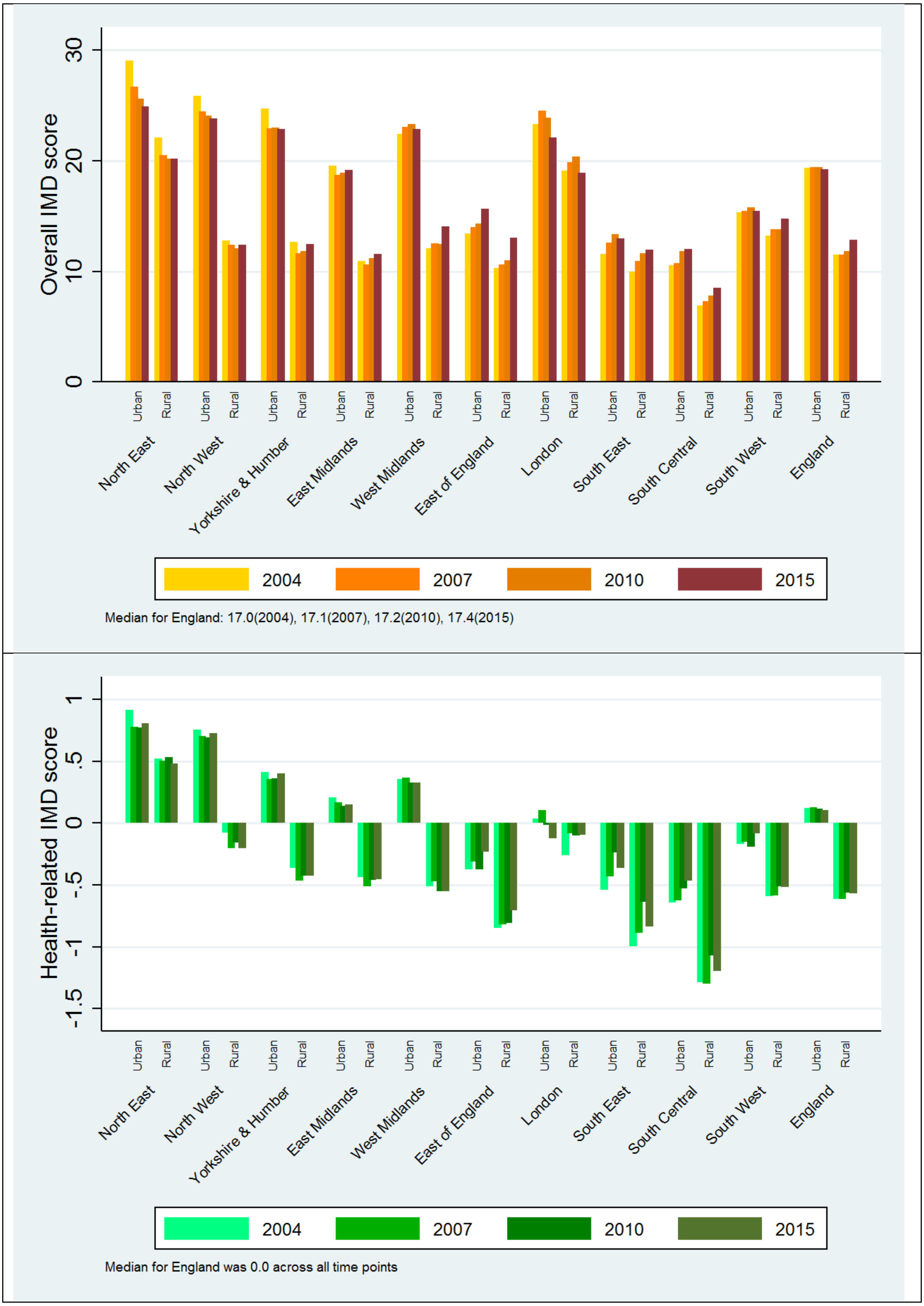

Supplement: Supplementary file 1 [file jech-2017-209895supp001.pdf]
