# Supplementary material for: Increasing socioeconomic gap between the young and old: temporal trends in health and overall deprivation in England by age, sex, urbanity and ethnicity, 2004–2015
Source: J Epidemiol Community Health. 2018 Mar 19;72(7):636–44. doi: 10.1136/jech-2017-209895 (PMC6031281; doi:10.1136/jech-2017-209895)
Supplement: Supplementary file 2 [file jech-2017-209895supp002.pdf]

Online appendix 2 for: “The increasing socio-economic gap between the young and old: temporal trends in health and overall deprivation in England by age, sex, urbanity and ethnicity, 2004 to 2015”

Table B1: Index of multiple deprivation domains, 2015

Income	• Adults and children in Income Support families<br>• Adults and children in income-based Jobseeker’s Allowance families<br>• Adults and children in income-based Employment and Support Allowance families<br>• Adults and children in Pension Credit (Guarantee) families<br>• Adults and children in Child Tax Credit and Working Tax Credit families, below 60% median income not already counted<br>• Asylum seekers in England in receipt of subsistence support, accommodation support, or both
Employment	• Claimants of Jobseeker’s Allowance, aged 18-59/64<br>• Claimants of Employment and Support Allowance, aged 18-59/64<br>• Claimants of Incapacity Benefit, aged 18-59/64<br>• Claimants of Severe Disablement Allowance, aged 18-59/64<br>• Claimants of Carer’s Allowance, aged 18-59/64
Health and disability	• Years of potential life lost<br>• Comparative illness and disability ratio<br>• Acute morbidity<br>• Mood and anxiety disorders
Education, Skills & Training	• Key stage 2 attainment: average points score<br>• Key stage 4 attainment: average points score<br>• Secondary school absence<br>• Staying on in education post 16<br>• Entry to higher education<br>• Adults with no or low qualifications, aged 25-59/64<br>• English language proficiency, aged 25-59/64
Crime	• Recorded crime rates for: Violence; Burglary; Theft; Criminal damage
Barriers to Housing & Services	• Road distance to: post office; primary school; general store / supermarket; GP surgery<br>• Household overcrowding<br>• Homelessness<br>• Housing affordability
Living Environment	• Housing in poor condition<br>• Houses without central heating<br>• Air quality<br>• Road traffic accidents

*Details available in the 2015 technical report of the English Indices of Deprivation:
https://www.gov.uk/government/publications/english-indices-of-deprivation-2015-technical-report

Figure B1: Percentage of White British, 2011 census*

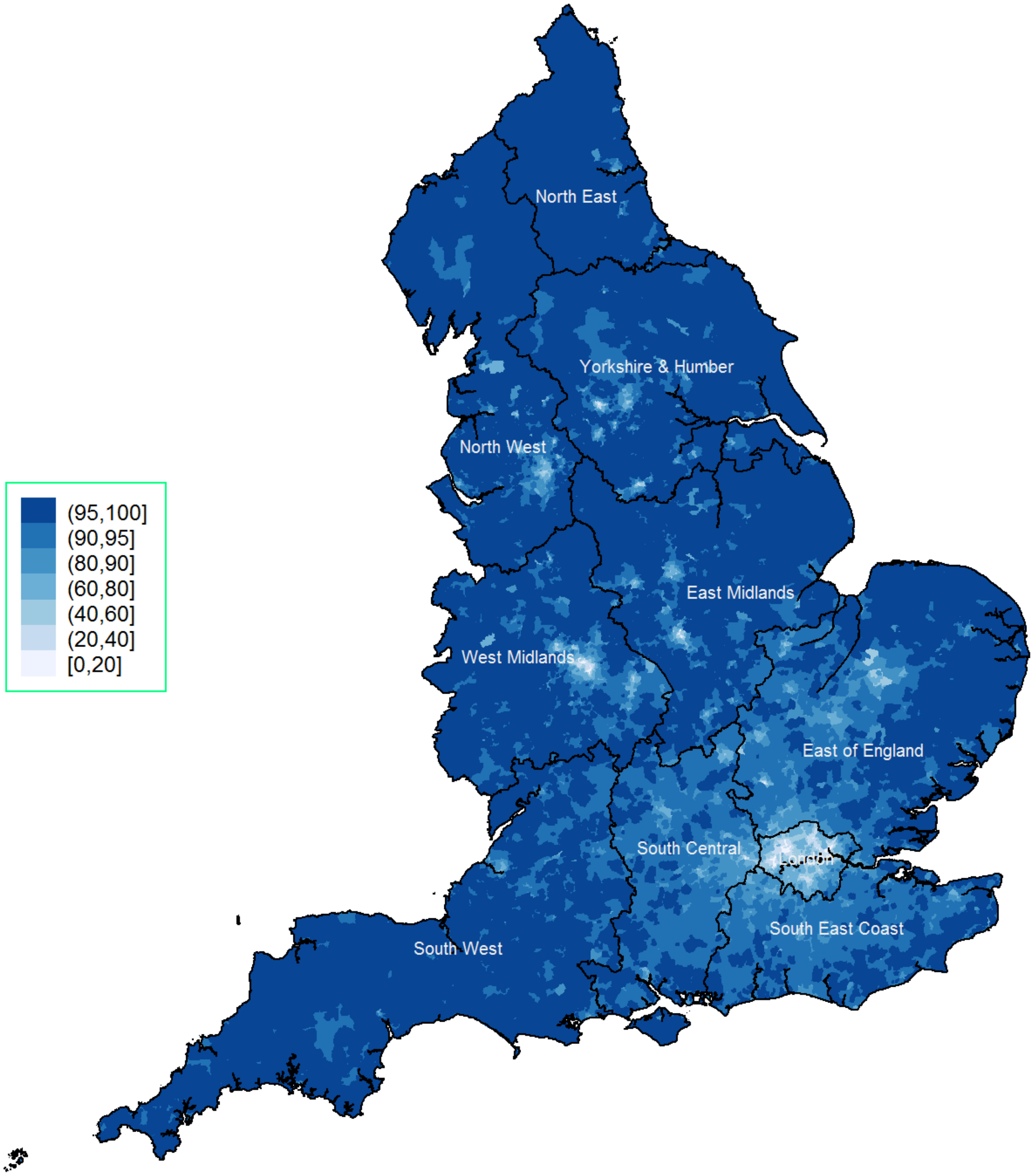

* The mean percentage of White British (across all LSOAs) was 91.5%

Figure B2: Distributions of the 2004 & 2015 Index of Multiple Deprivation (IMD) for all English Lower Super Output Areas

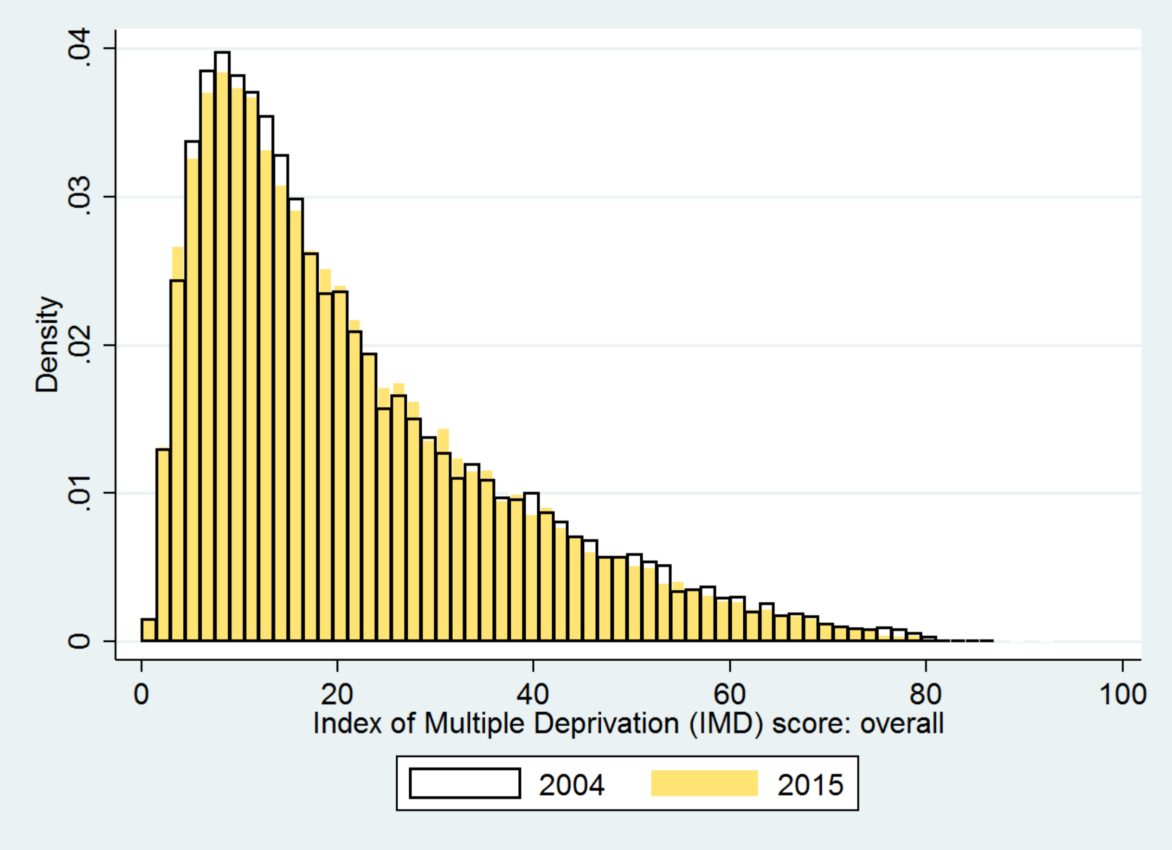

Figure B3: Distributions of the 2004 & 2015 health domain of the IMD for all English Lower Super Output Areas

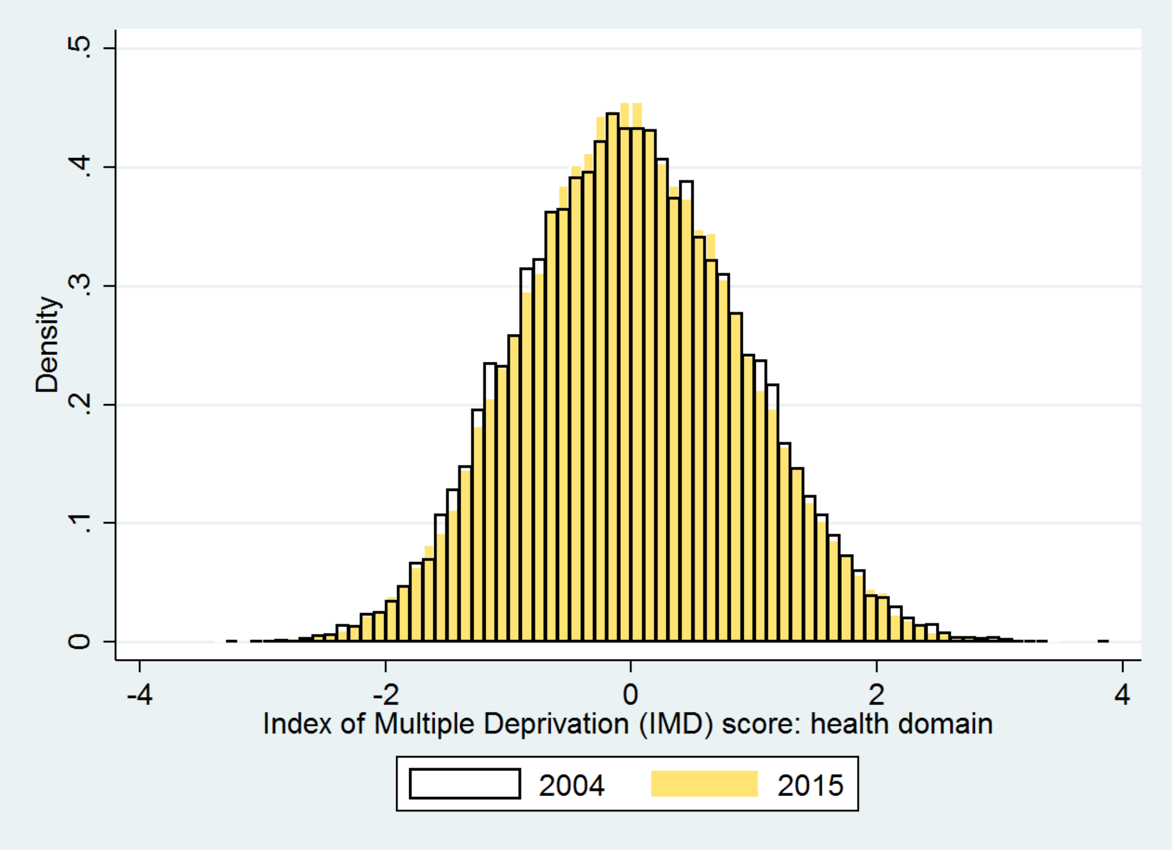

Table B2: Summary statistics for the 2015 Index of Multiple Deprivation and its health domain

Index of Multiple Deprivation				
	Percentiles	Smallest		
1%	2.28	0.48		
5%	4.09	0.53		
10%	5.67	0.57	Observations	32844
25%	9.65	0.58		
50%	17.40		Mean	21.67
		Largest	Std. Dev.	15.59
75%	30.07	85.62		
90%	44.57	86.44	Variance	243.17
95%	53.69	88.52	Skewness	1.13
99%	68.81	92.60	Kurtosis	3.85
Health domain IMD				
	Percentiles	Smallest		
1%	-2.00	-3.33		
5%	-1.44	-3.25		
10%	-1.13	-2.98	Observations	32844
25%	-0.61	-2.95		
50%	-0.02		Mean	0.00
		Largest	Std. Dev.	0.89
75%	0.60	3.13		
90%	1.16	3.16	Variance	0.79
95%	1.50	3.29	Skewness	0.08
99%	2.08	3.46	Kurtosis	2.93

Table B3: Centiles for the 2015 Index of Multiple Deprivation and its health domain

	Overall IMD		health domain IMD	
centile	from	to	from	to
1	0.477	2.279	-3.329	-2.004
2	2.281	2.882	-2.003	-1.781
3	2.884	3.321	-1.779	-1.645
4	3.322	3.744	-1.644	-1.532
5	3.746	4.086	-1.531	-1.436
6	4.087	4.409	-1.435	-1.356
7	4.41	4.752	-1.355	-1.291
8	4.754	5.079	-1.29	-1.231
9	5.08	5.392	-1.23	-1.185
10	5.394	5.673	-1.184	-1.134
11	5.674	5.958	-1.133	-1.088
12	5.959	6.222	-1.087	-1.042
13	6.223	6.516	-1.041	-1.002
14	6.517	6.792	-1.001	-0.961
15	6.793	7.04	-0.96	-0.926
16	7.041	7.327	-0.925	-0.89
17	7.328	7.589	-0.889	-0.856
18	7.59	7.845	-0.855	-0.821
19	7.847	8.112	-0.82	-0.787
20	8.113	8.372	-0.786	-0.755
21	8.373	8.624	-0.754	-0.725
22	8.625	8.863	-0.724	-0.691
23	8.864	9.124	-0.69	-0.663
24	9.125	9.398	-0.662	-0.636
25	9.399	9.652	-0.635	-0.611
26	9.657	9.94	-0.61	-0.583
27	9.941	10.181	-0.582	-0.557
28	10.182	10.463	-0.556	-0.533
29	10.464	10.738	-0.532	-0.507
30	10.739	11.016	-0.506	-0.48
31	11.018	11.279	-0.479	-0.455
32	11.28	11.538	-0.454	-0.432
33	11.539	11.822	-0.431	-0.407
34	11.825	12.101	-0.406	-0.382
35	12.102	12.391	-0.381	-0.357
36	12.392	12.689	-0.356	-0.333
37	12.69	12.982	-0.332	-0.309
38	12.983	13.3	-0.308	-0.284
39	13.301	13.612	-0.283	-0.261
40	13.613	13.922	-0.26	-0.24
41	13.924	14.266	-0.239	-0.218
42	14.267	14.581	-0.217	-0.196

	Overall IMD		health domain IMD	
centile	from	to	from	to
43	14.583	14.919	-0.195	-0.175
44	14.924	15.244	-0.174	-0.153
45	15.245	15.576	-0.152	-0.131
46	15.577	15.888	-0.13	-0.106
47	15.89	16.268	-0.105	-0.085
48	16.269	16.627	-0.084	-0.064
49	16.628	17.006	-0.063	-0.044
50	17.01	17.398	-0.043	-0.02
51	17.399	17.762	-0.019	0.003
52	17.763	18.139	0.004	0.024
53	18.14	18.538	0.025	0.047
54	18.542	18.937	0.048	0.068
55	18.939	19.312	0.069	0.091
56	19.315	19.779	0.092	0.113
57	19.781	20.141	0.114	0.137
58	20.142	20.555	0.138	0.159
59	20.558	21	0.16	0.182
60	21.001	21.432	0.183	0.206
61	21.434	21.904	0.207	0.231
62	21.906	22.379	0.232	0.256
63	22.381	22.862	0.257	0.281
64	22.863	23.359	0.282	0.305
65	23.36	23.905	0.306	0.331
66	23.906	24.466	0.332	0.355
67	24.471	25.046	0.356	0.381
68	25.048	25.619	0.382	0.408
69	25.62	26.196	0.409	0.434
70	26.199	26.751	0.435	0.462
71	26.754	27.375	0.463	0.487
72	27.377	27.948	0.488	0.519
73	27.949	28.607	0.52	0.546
74	28.614	29.337	0.547	0.574
75	29.339	30.066	0.575	0.603
76	30.068	30.741	0.604	0.632
77	30.744	31.433	0.633	0.661
78	31.437	32.236	0.662	0.691
79	32.239	33.05	0.692	0.721
80	33.056	33.876	0.722	0.752
81	33.881	34.771	0.753	0.786
82	34.772	35.61	0.787	0.82
83	35.613	36.559	0.821	0.859
84	36.571	37.616	0.86	0.894

	Overall IMD		health domain IMD	
centile	from	to	from	to
85	37.619	38.591	0.895	0.933
86	38.594	39.669	0.934	0.974
87	39.674	40.826	0.975	1.017
88	40.836	41.907	1.018	1.064
89	41.912	43.163	1.065	1.112
90	43.174	44.572	1.113	1.162
91	44.574	46.091	1.163	1.222
92	46.094	47.861	1.223	1.281
93	47.867	49.541	1.282	1.344
94	49.545	51.479	1.345	1.413
95	51.481	53.694	1.414	1.5
96	53.703	56.215	1.501	1.599
97	56.222	59.239	1.601	1.714
98	59.258	63.482	1.715	1.866
99	63.494	68.811	1.867	2.078
100	68.816	92.601	2.079	3.458
